# Supplementary material for: Association between ambient temperature and hypertensive disorders in pregnancy in China
Source: Nat Commun. 2020 Jun 10;11:2925. doi: 10.1038/s41467-020-16775-8 (PMC7286884; doi:10.1038/s41467-020-16775-8)
Supplement: Supplementary file 1 — Supplementary Information [file 41467_2020_16775_MOESM1_ESM.pdf]

Supplementary Information

Title: Association between ambient temperature and hypertensive disorders in pregnancy in China

Authors: Xiong et al.

Included Files

Supplementary Fig. 1-16

Supplementary Table 1

Supplementary Figures

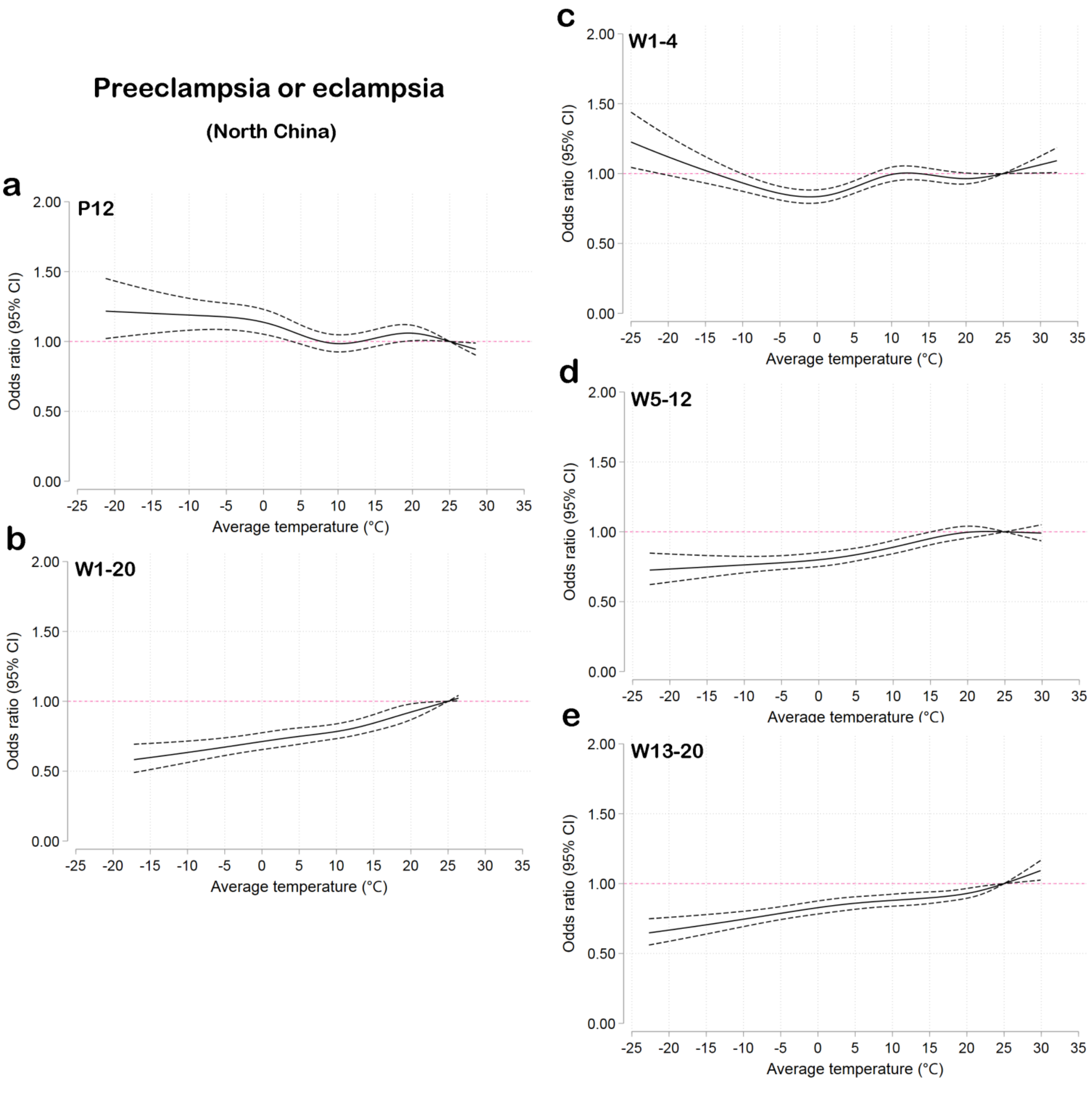

Supplementary Fig. 1 Curves for average temperature and preeclampsia or eclampsia in North China. Solid black lines corresponding to points estimate of aORs of preeclampsia or eclampsia; dashed black lines correspond to 95% confidence intervals. **(a)** 12 weeks preconception; **(b)** 1-20 weeks postconception; **(c)** 1-4 weeks postconception; **(d)** 5-12 weeks postconception; **(e)** 13-20 weeks postconception. Abbreviations: P, preconception; W, weeks postconception. Source data are provided as a Source Data file.

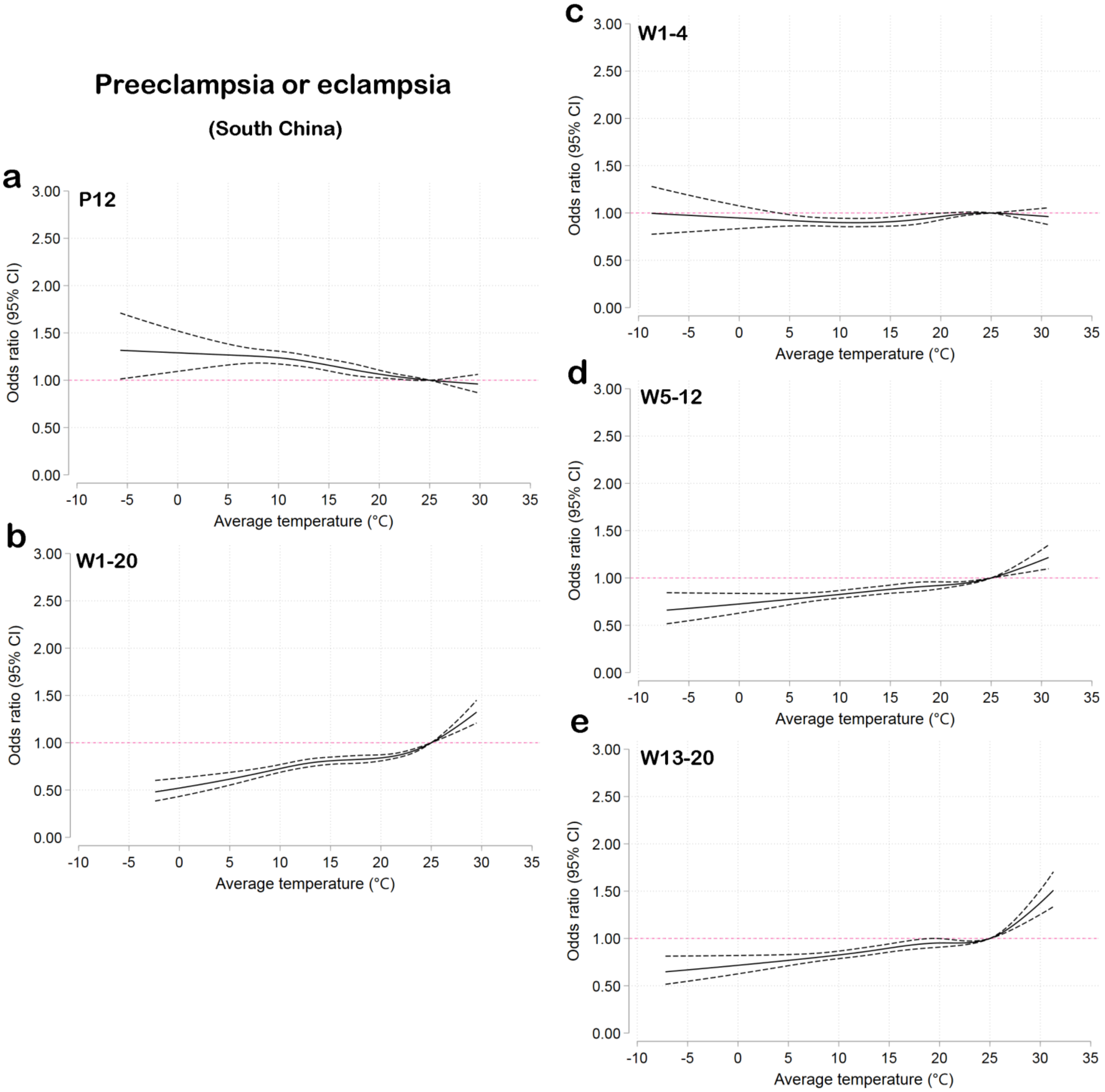

Supplementary Fig. 2 Curves for average temperature and preeclampsia or eclampsia in South China. Solid black lines corresponding to points estimate of aORs of preeclampsia or eclampsia; dashed black lines correspond to 95% confidence intervals. **(a)** 12 weeks preconception; **(b)** 1-20 weeks postconception; **(c)** 1-4 weeks postconception; **(d)** 5-12 weeks postconception; **(e)** 13-20 weeks postconception. Abbreviations: P, preconception; W, weeks postconception. Source data are provided as a Source Data file.

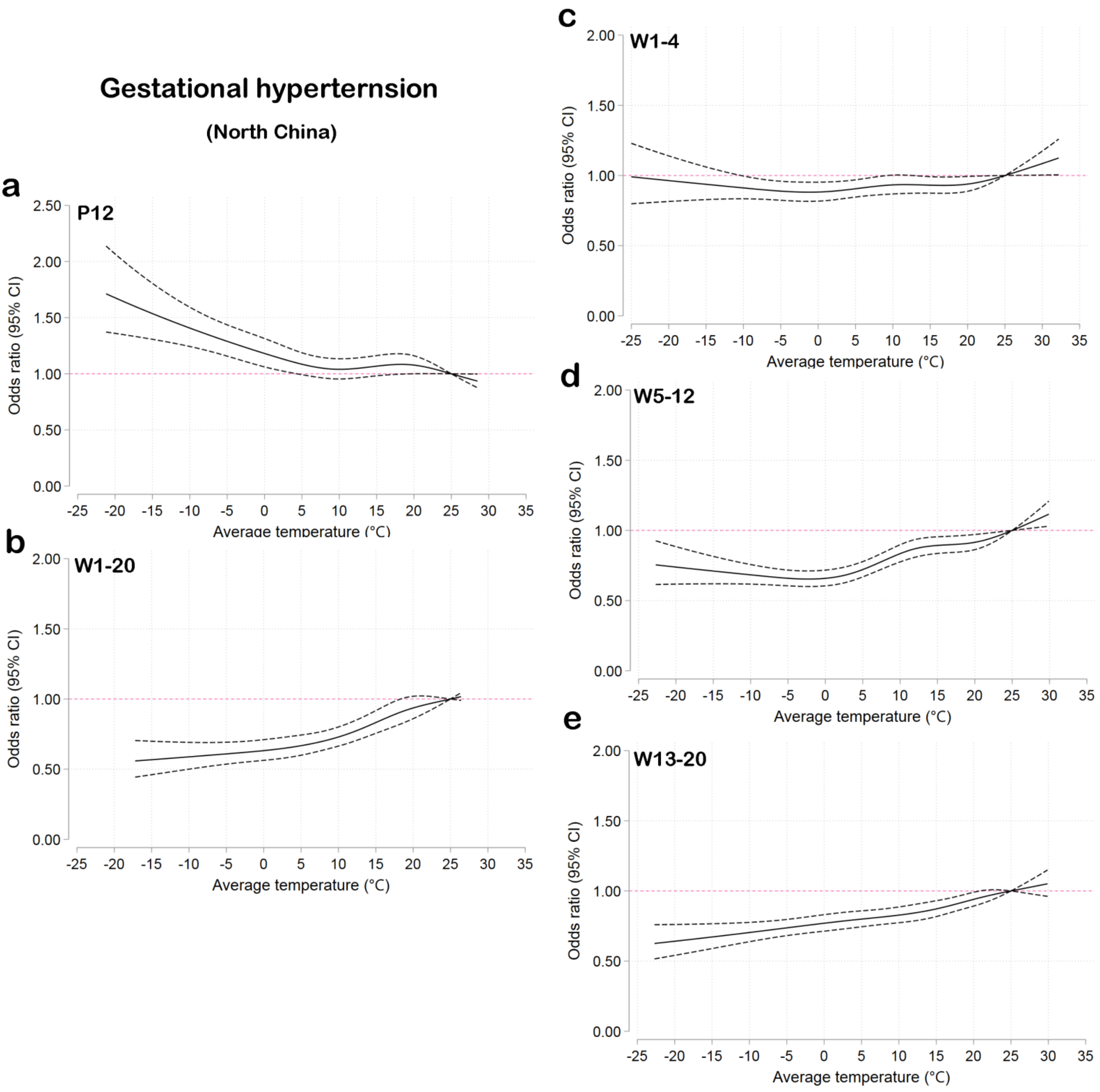

Supplementary Fig. 3 Curves for average temperature and gestational hypertension in North China. Solid black lines correspond to points estimate of aORs of gestational hypertension; dashed black lines correspond to 95% confidence intervals. **(a)** 12 weeks preconception; **(b)** 1-20 weeks postconception; **(c)** 1-4 weeks postconception; **(d)** 5-12 weeks postconception; **(e)** 13-20 weeks postconception. Abbreviations: P, preconception; W, weeks postconception. Source data are provided as a Source Data file.

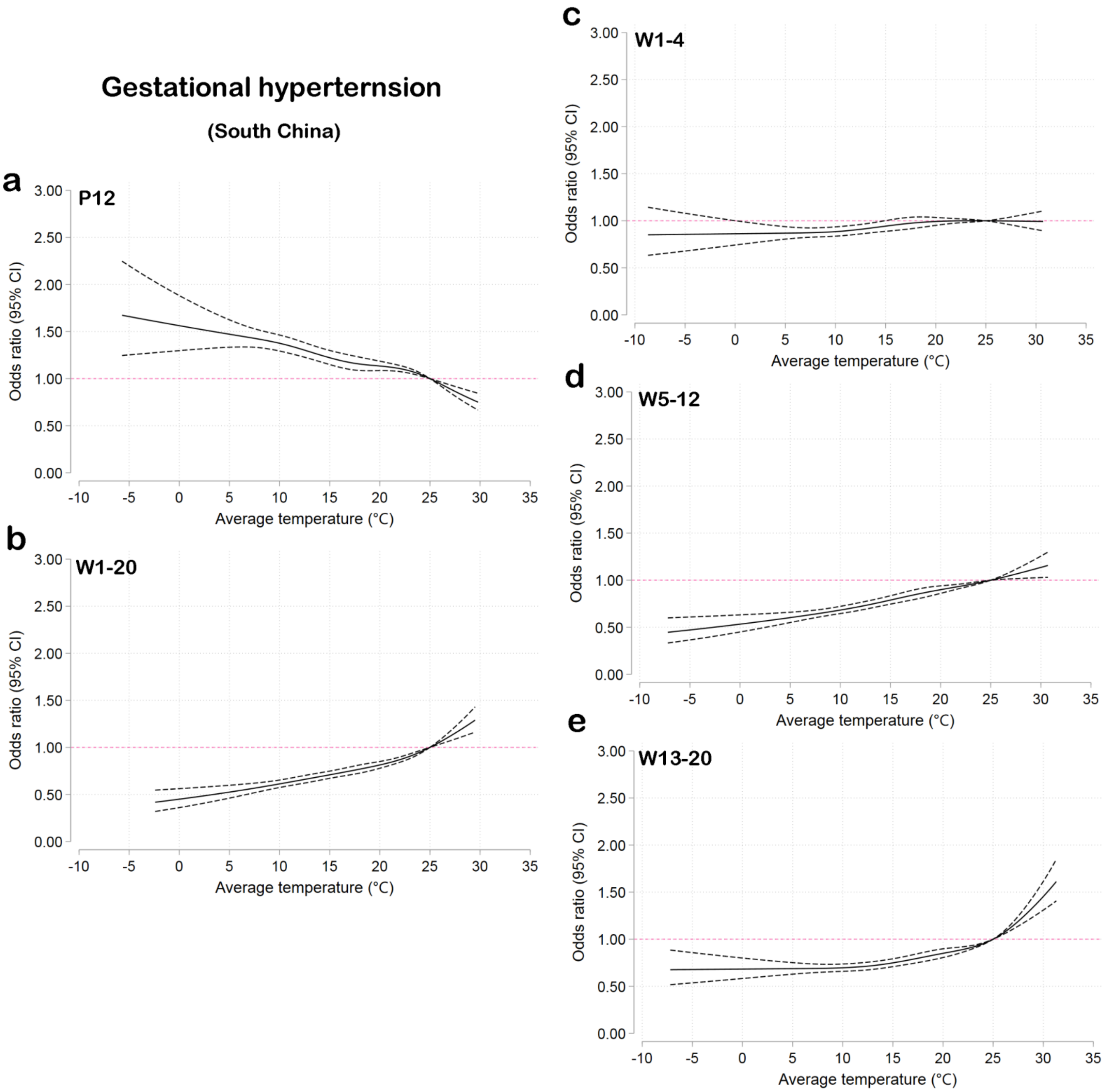

Supplementary Fig. 4 Curves for average temperature and gestational hypertension in South China. Solid black lines correspond to points estimate of aORs of gestational hypertension; dashed black lines correspond to 95% confidence intervals. **(a)** 12 weeks preconception; **(b)** 1-20 weeks postconception; **(c)** 1-4 weeks postconception; **(d)** 5-12 weeks postconception; **(e)** 13-20 weeks postconception. Abbreviations: P, preconception; W, weeks postconception. Source data are provided as a Source Data file.

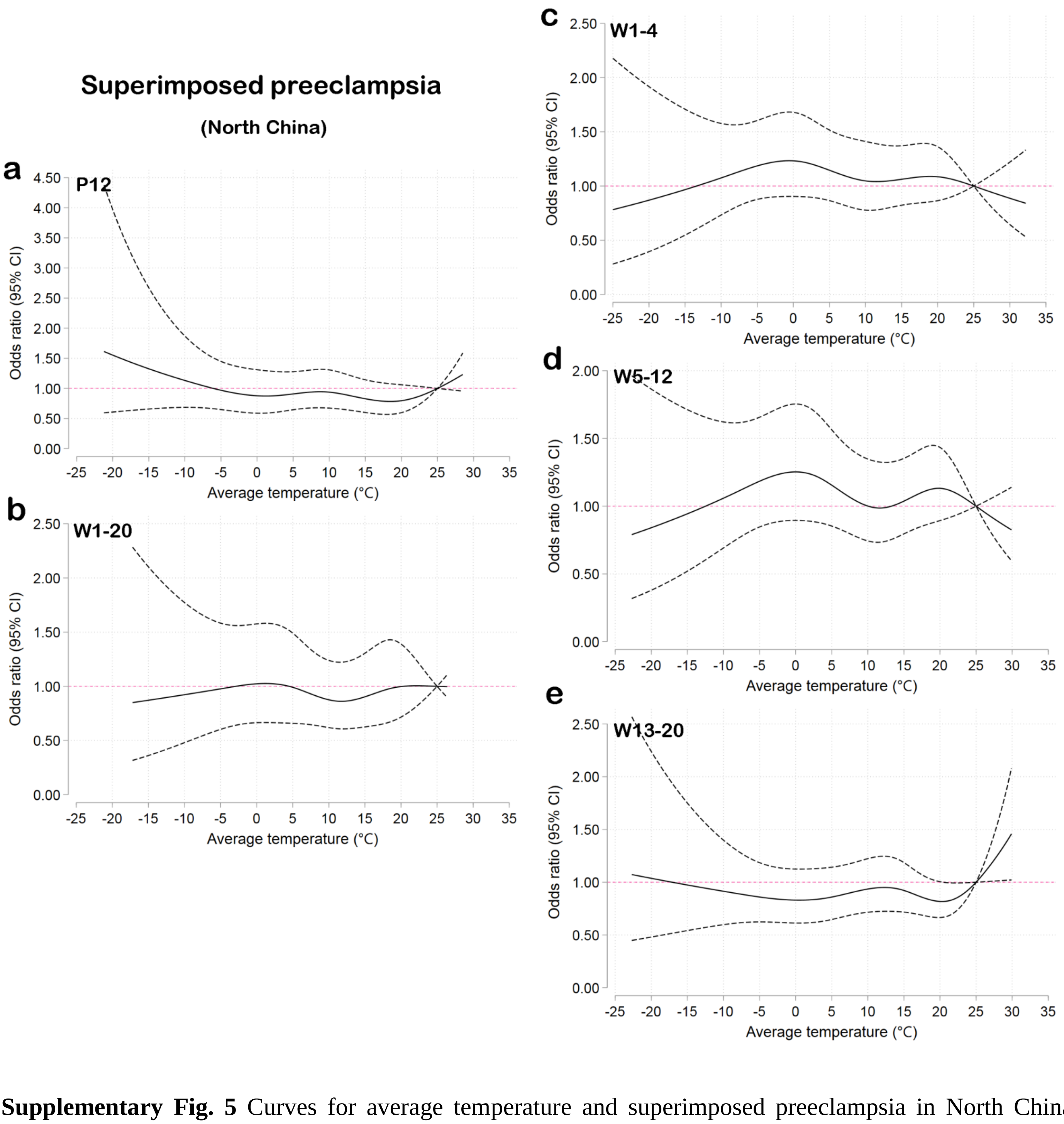

Supplementary Fig. 5 Curves for average temperature and superimposed preeclampsia in North China. Solid black lines correspond to points estimate of aORs of superimposed preeclampsia; dashed black lines correspond to 95% confidence intervals. **(a)** 12 weeks preconception; **(b)** 1-20 weeks postconception; **(c)** 1-4 weeks postconception; **(d)** 5-12 weeks postconception; **(e)** 13-20 weeks postconception. Abbreviations: P, preconception; W, weeks postconception. Source data are provided as a Source Data file.

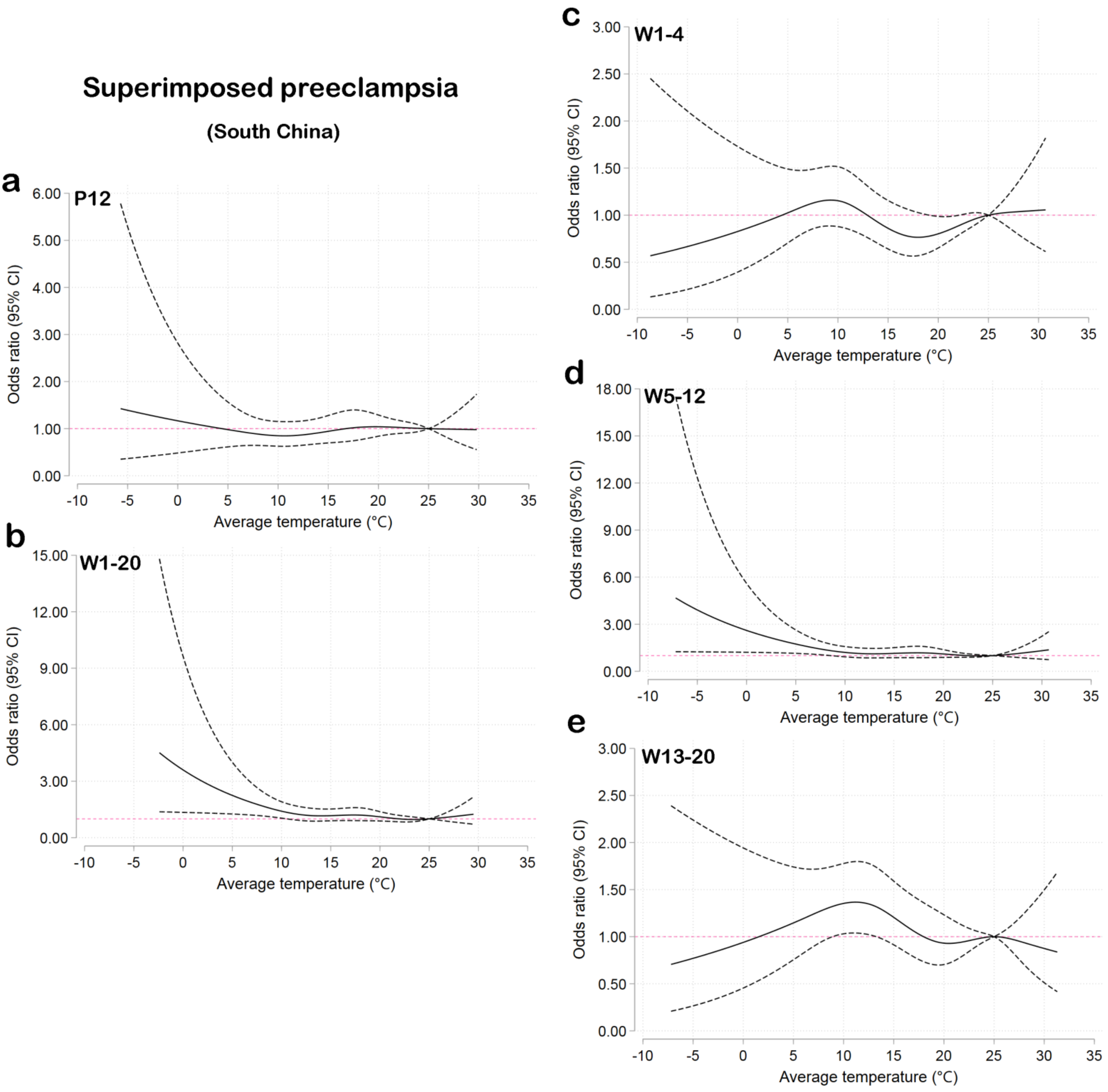

Supplementary Fig. 6 Curves for average temperature and superimposed preeclampsia in South China. Solid black lines correspond to points estimate of aORs of superimposed preeclampsia; dashed black lines correspond to 95% confidence intervals. **(a)** 12 weeks preconception; **(b)** 1-20 weeks postconception; **(c)** 1-4 weeks postconception; **(d)** 5-12 weeks postconception; **(e)** 13-20 weeks postconception. Abbreviations: P, preconception; W, weeks postconception. Source data are provided as a Source Data file.

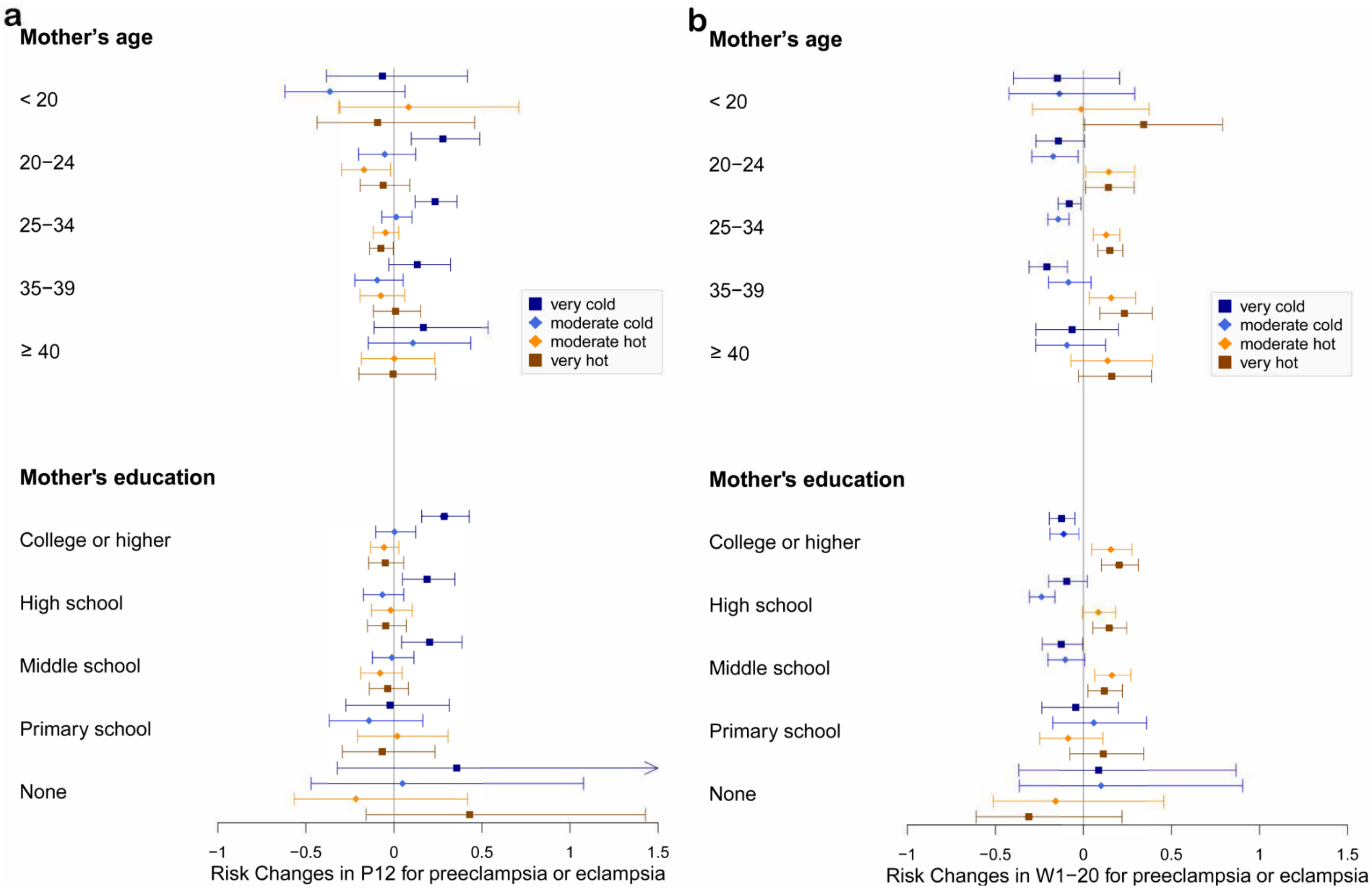

Supplementary Fig. 7 Risk change in preeclampsia or eclampsia to extreme temperature by maternal mother's age and education level. **(a)** 12 weeks preconception; **(b)** the first half of pregnancy. Data are presented as change in aORs with 95% confidence intervals. Error bars correspond to 95% confidence intervals, centre for the error bars correspond to point estimates of change in aORs. Source data are provided as a Source Data file.

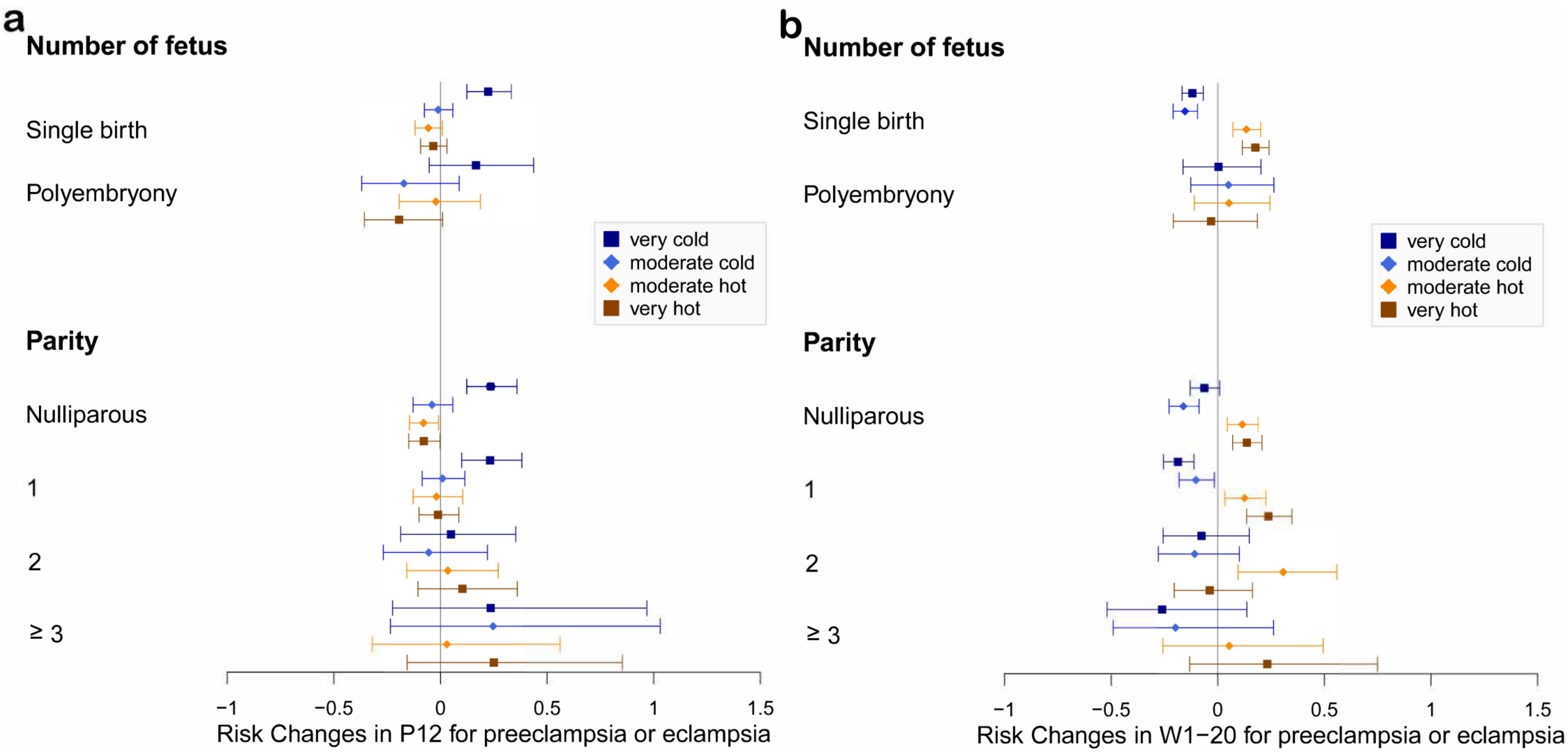

Supplementary Fig. 8 Risk change in preeclampsia or eclampsia to extreme temperature by number of fetuses and parity. **(a)** 12 weeks preconception; **(b)** the first half of pregnancy. Data are presented as change in aORs with 95% confidence intervals. Error bars correspond to 95% confidence intervals, centre for the error bars correspond to point estimates of change in aORs. Source data are provided as a Source Data file.

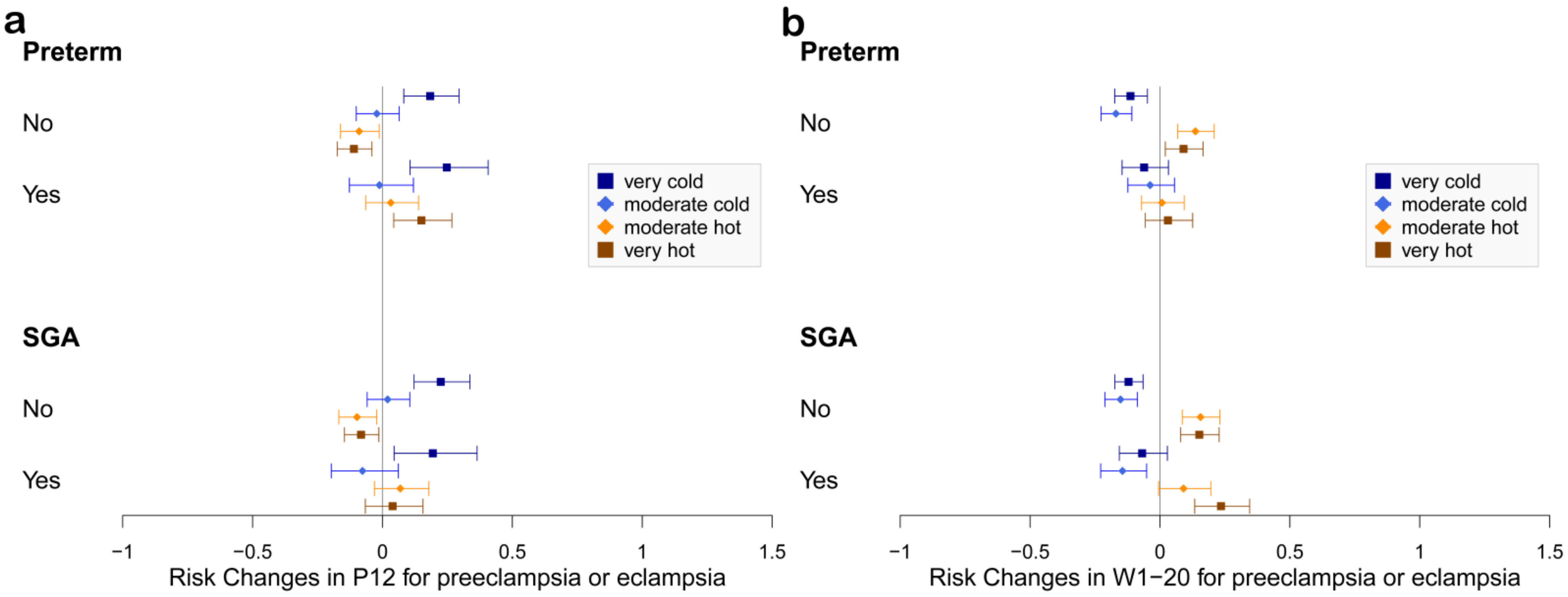

Supplementary Fig. 9 Risk change in preeclampsia or eclampsia to extreme temperature by preterm and small for gestational age infants. **(a)** 12 weeks preconception; **(b)** the first half of pregnancy. SGA: small for gestational age. Data are presented as change in aORs with 95% confidence intervals. Error bars correspond to 95% confidence intervals, centre for the error bars correspond to point estimates of change in aORs. Source data are provided as a Source Data file.

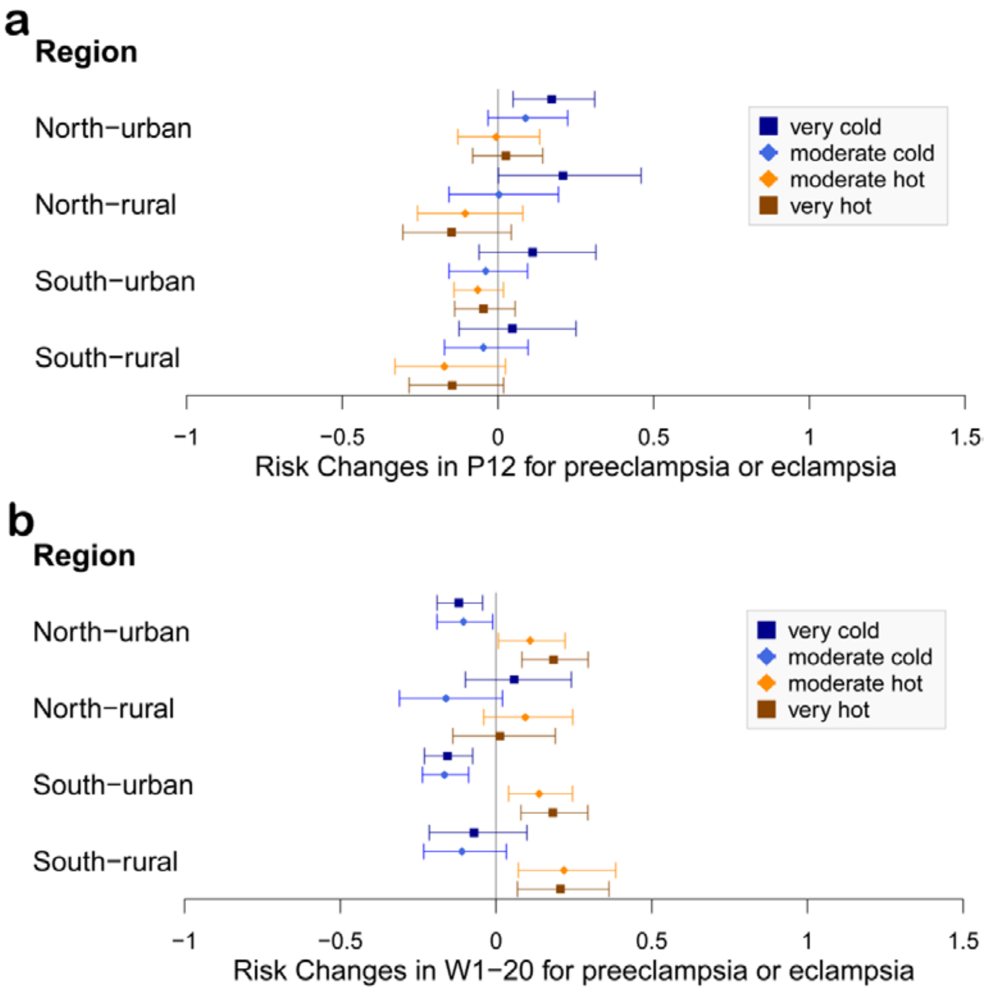

Supplementary Fig. 10 Risk change in preeclampsia or eclampsia to extreme temperature by region. **(a)** 12 weeks preconception; **(b)** the first half of pregnancy. Data are presented as change in aORs with 95% confidence intervals. Error bars correspond to 95% confidence intervals, centre for the error bars correspond to point estimates of change in aORs. Source data are provided as a Source Data file.

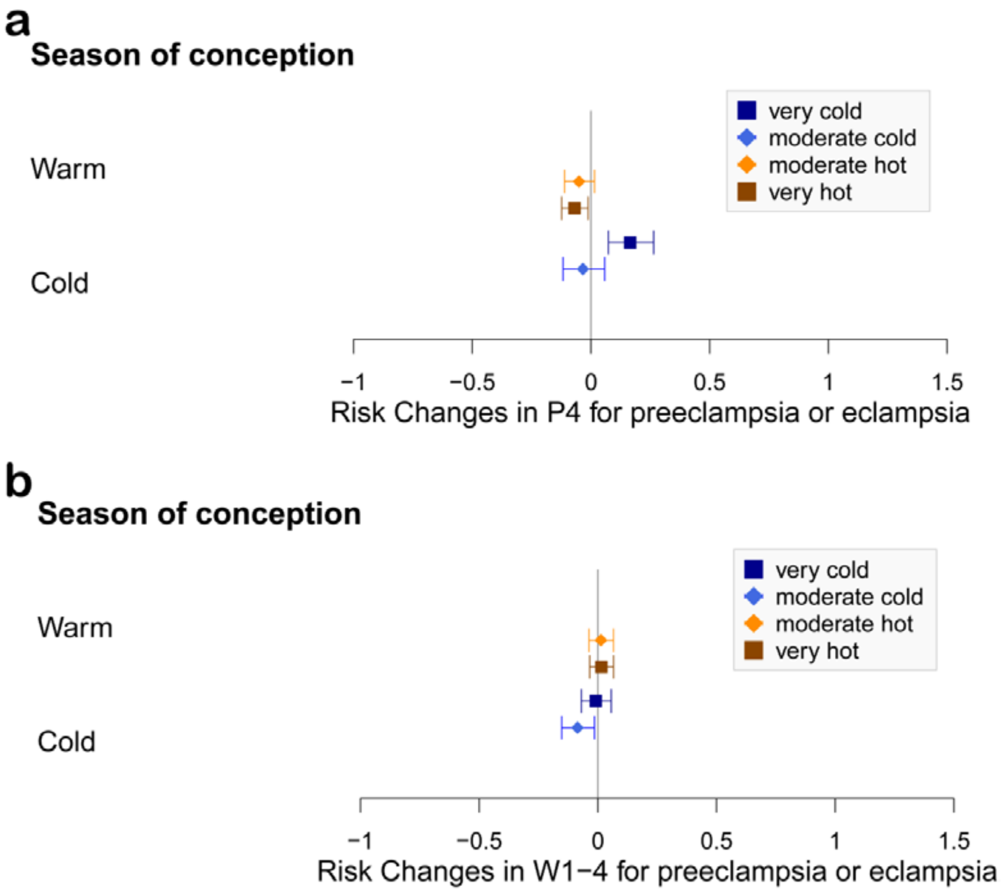

Supplementary Fig. 11 Risk change in preeclampsia or eclampsia to extreme temperature by season of conception. **(a)** 4 weeks preconception; **(b)** 1-4 weeks postconception. Data are presented as change in aORs with 95% confidence intervals. Error bars correspond to 95% confidence intervals, centre for the error bars correspond to point estimates of change in aORs. Source data are provided as a Source Data file.

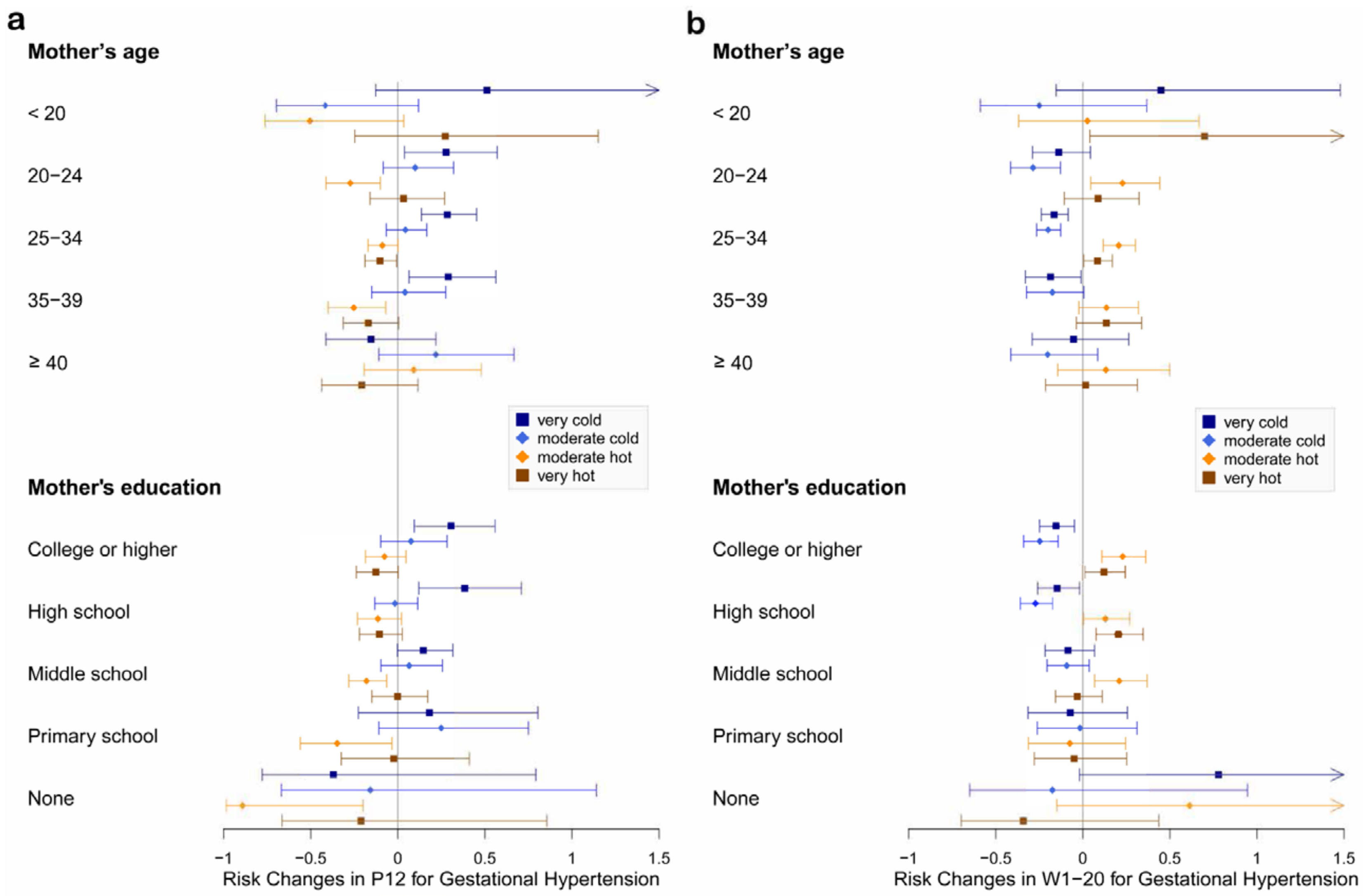

Supplementary Fig. 12 Risk change in gestational hypertension to extreme temperature by maternal mother's age and education level. **(a)** 12 weeks preconception; **(b)** the first half of pregnancy. Data are presented as change in aORs with 95% confidence intervals. Error bars correspond to 95% confidence intervals, centre for the error bars correspond to point estimates of change in aORs. Source data are provided as a Source Data

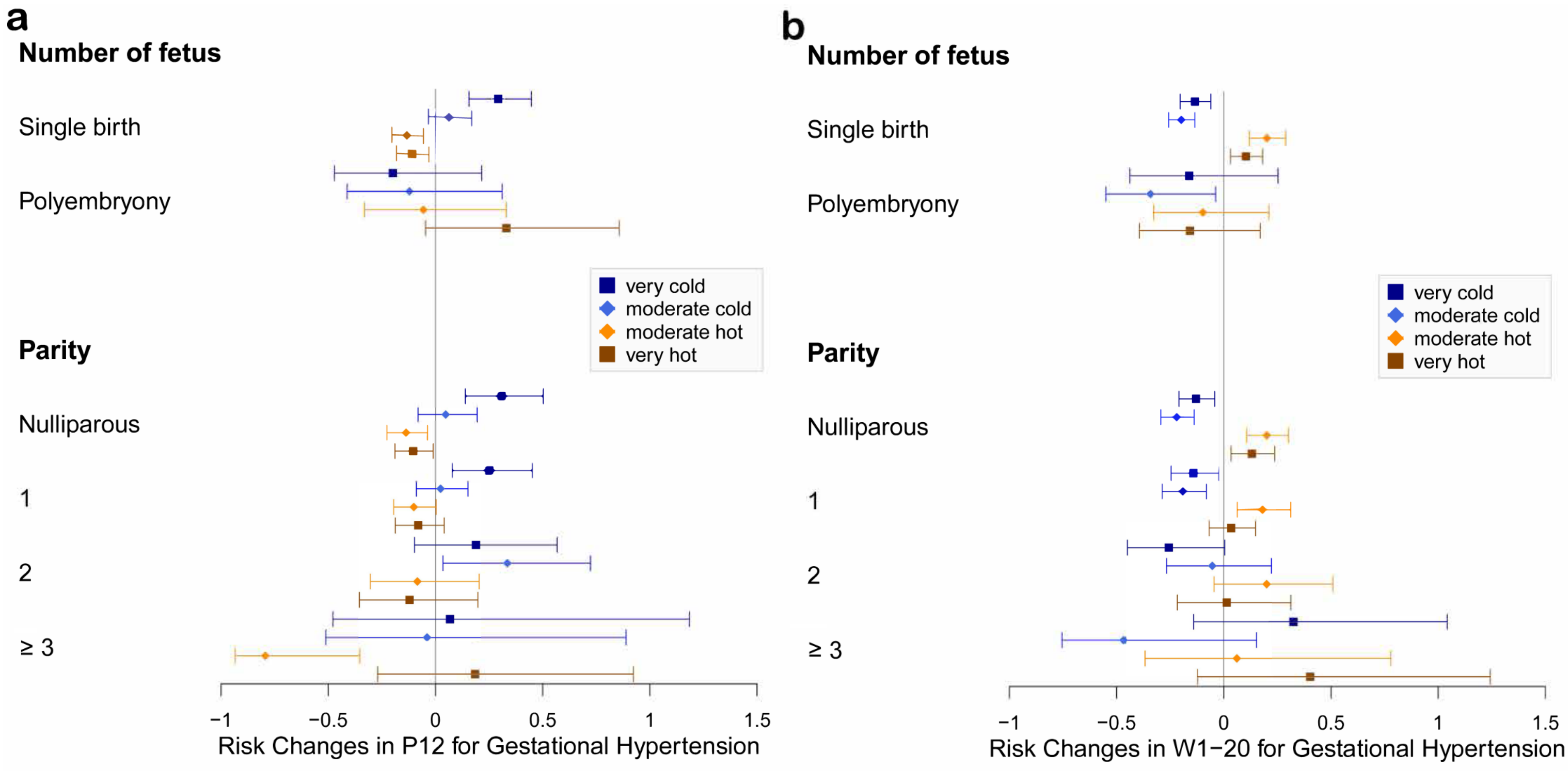

Supplementary Fig. 13 Risk change in gestational hypertension to extreme temperature by number of fetuses and parity. **(a)** 12 weeks preconception; **(b)** the first half of pregnancy. Data are presented as change in aORs with 95% confidence intervals. Error bars correspond to 95% confidence intervals, centre for the error bars correspond to point estimates of change in aORs. Source data are provided as a Source Data file.

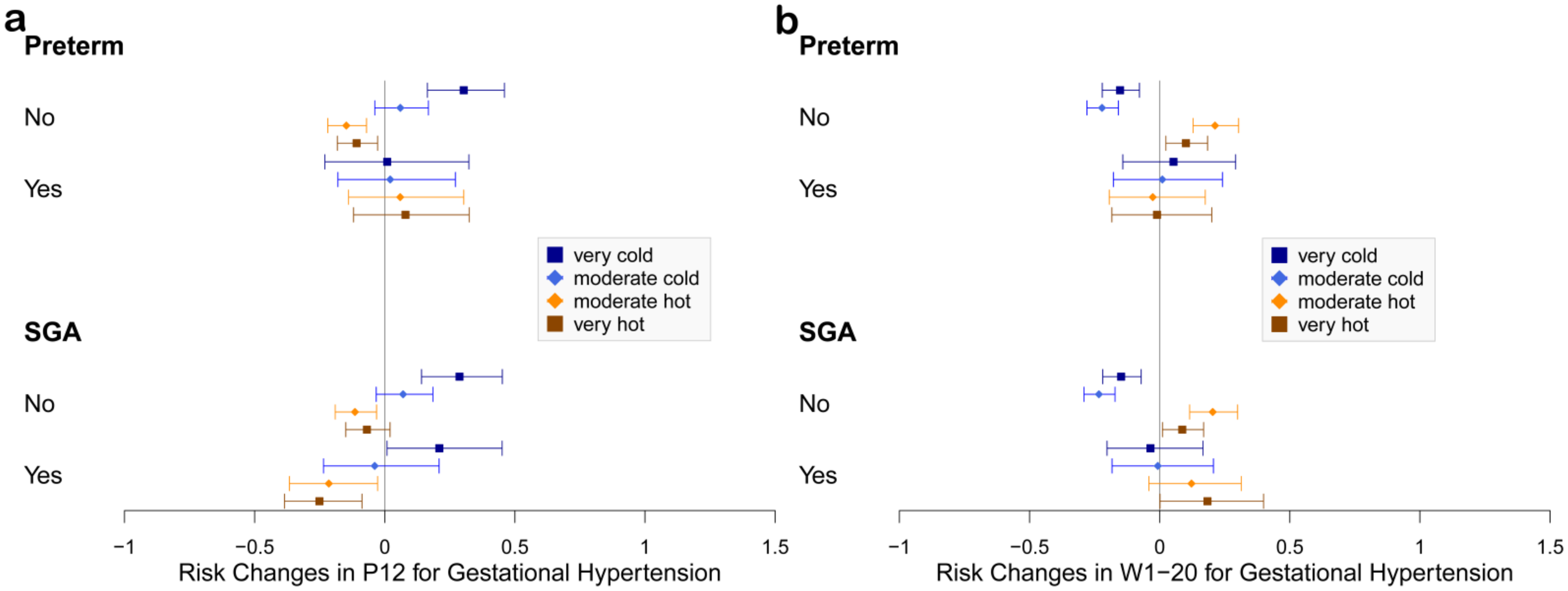

Supplementary Fig. 14 Risk change in gestational hypertension to extreme temperature by preterm and small for gestational age infants. **(a)** 12 weeks preconception; **(b)** the first half of pregnancy. SGA: small for gestational age. Data are presented as change in aORs with 95% confidence intervals. Error bars correspond to 95% confidence intervals, centre for the error bars correspond to point estimates of change in aORs. Source data are provided as a Source Data file.

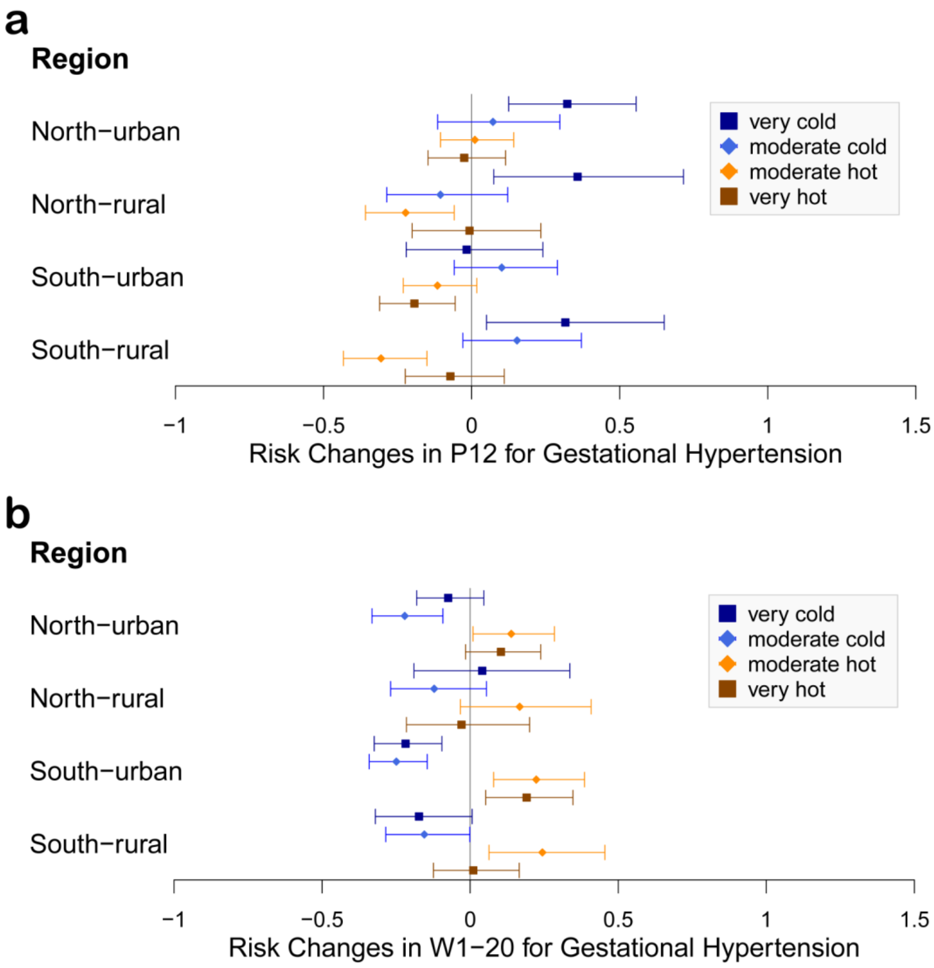

Supplementary Fig. 15 Risk change in gestational hypertension to extreme temperature by region. **(a)** 12 weeks preconception; **(b)** the first half of pregnancy. Data are presented as change in aORs with 95% confidence intervals. Error bars correspond to 95% confidence intervals, centre for the error bars correspond to point estimates of change in aORs. Source data are provided as a Source Data file.

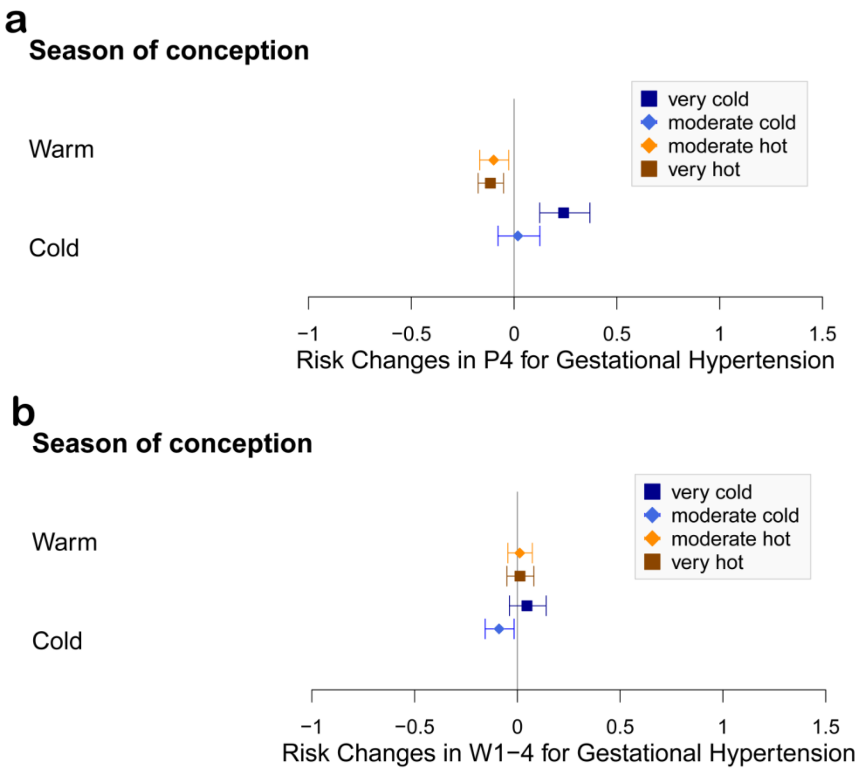

Supplementary Fig. 16 Risk change in gestational hypertension to extreme temperature by season of conception. **(a)** 4 weeks preconception; **(b)** 1-4 weeks postconception. Data are presented as change in aORs with 95% confidence intervals. Error bars correspond to 95% confidence intervals, centre for the error bars correspond to point estimates of change in aORs. Source data are provided as a Source Data file.

Supplementary Table 1 Stratified variables and covariates in subgroup analyses.

Stratified character	Covariates
Region(north-rural, north-urban, south-rural, south-urban)	region(west, central and east), hospital level, antenatal care visits, marital status, mother's education, mother's age, parity, number of fetus, elevation, humidity, and air pollution exposure
Mother's education	hospital level, antenatal care visits, marital status, region(west, central and east), mother's age, parity, number of fetus, elevation, humidity, and air pollution exposure
Mother's age	hospital level, antenatal care visits, marital status, region(west, central and east), mother's education, parity, number of fetus, elevation, humidity, and air pollution exposure
Parity	hospital level, antenatal care visits, marital status, region(west, central and east), mother's education, mother's age, number of fetus, elevation, humidity, and air pollution exposure
Number of fetus	hospital level, antenatal care visits, marital status, region(west, central and east), mother's education, mother's age, parity, elevation, humidity, and air pollution exposure
Preterm	hospital level, antenatal care visits, marital status, region(west, central and east), mother's education, mother's age, parity, number of fetus, elevation, humidity, and air pollution exposure
Small for gestational age	hospital level, antenatal care visits, marital status, region(west, central and east), mother's education, mother's age, parity, number of fetus, elevation, humidity, and air pollution exposure
Season of conception	hospital level, antenatal care visits, marital status, region(west, central and east), mother's education, mother's age, parity, number of fetus, elevation, humidity, and air pollution exposure
