# Supplementary material for: Association between ambient temperature and hypertensive disorders in pregnancy in China
Source: Nat Commun. 2020 Jun 10;11:2925. doi: 10.1038/s41467-020-16775-8 (PMC7286884; doi:10.1038/s41467-020-16775-8)
Supplement: Supplementary file 3 — Description of Additional Supplementary Files [file 41467_2020_16775_MOESM3_ESM.pdf]

Description of Additional Supplementary Files

File Name: Supplementary Software 1
Description: Codes used for the statistical models (including a Stata code file of RCS analysis for average temperature analysis, a SAS code file for extreme temperature analysis, a R code file for drawing forest plot of subgroup analysis results, and an introduction file of variables in original datasets).
