# Supplementary material for: Association between ambient temperature and hypertensive disorders in pregnancy in China
Source: Nat Commun. 2020 Jun 10;11:2925. doi: 10.1038/s41467-020-16775-8 (PMC7286884; doi:10.1038/s41467-020-16775-8)
Supplement: Supplementary file 4 — Supplementary Software 1 [file 41467_2020_16775_MOESM4_ESM.zip › appendix. variable explanation for code.docx]

The do file in the supplement files includes the source codes for restricted cubic spline line (RCS) analysis and plots. The “xblc” package of Stata is needed (typing “ssc install xblc”in the command window of Stata). The do file runs well in the Stata 16.0 with Windows 7.

The sas file in the supplement files includes the source codes for relative temperature exposure analysis using the logistic regression. The sas file runs well in the SAS 9.4 with Windows 7. The R code was used to draw the forest plot using the package of "forestplot".

Varaibles used in models:

| region | a category variable. 1: east, 2: central, and 3: west |
| --- | --- |
| hosp_level | Hospital level, a category variable. 1: level 1, 2: level 2, and 3: level 3 |
| pre_visit | Antenatal care visits, a category variable. 0: 0 times, 1: 1-3 times, 2: 4-6 times, 3: 7-9 times, 4: >= 10 times |
| edu | Mother's education, a category variable. 1: college of higher, 2: high school, 3: middle school, 4: primary school, 5: none |
| marry | Marital status, a category variable. 1: single/widower/divorced/cohabitation, 2: married |
| age_cat | Mother's age, a category variable. 1: <20 yrs, 2: 20-24 yrs, 3: 25-34 yrs, 4: 35-39 yrs, 5: >= 40 yrs |
| preg_time | Parity, a category variable. 0: nulliparous, 1: 1 time , 2: 2 times, 3: >= 3 times |
| b_21 | Number of fetus, a category variable. 1: single birth, 2: polyembryony |
| c_elevation | Elevalition of the hospital location. 1: < 800 meters, 2: 800-<1800 meters, 3: 1800-<2800 meters, 4: >= 2800 meters |
| m_12 | Average temperature exposure between 1-12 weeks of preconception. |
| r_12 | Average humidity exposure between 1-12 weeks of preconception. |
| a_12 | Average AQI exposure between 1-12 weeks of preconception. |
| m_4 | Average temperature exposure between 1-4 weeks of preconception. |
| r_4 | Average humidity exposure between 1-4 weeks of preconception. |
| a_4 | Average AQI exposure between 1-4 weeks of preconception. |
| m14 | Average temperature exposure between 1-4 weeks of gestation. |
| r14 | Average humidity exposure between 1-4 weeks of gestation. |
| a14 | Average AQI exposure between 1-4 weeks of gestation. |
| m512 | Average temperature exposure between 5-12 weeks of gestation. |
| r512 | Average humidity exposure between 5-12 weeks of gestation. |
| a512 | Average AQI exposure between 5-12 weeks of gestation. |
| m1320 | Average temperature exposure between 13-20 weeks of gestation. |
| r1320 | Average humidity exposure between 13-20 weeks of gestation. |
| a1320 | Average AQI exposure between 13-20 weeks of gestation. |
| m20 | Average temperature exposure between 1-20 weeks of gestation. |
| r20 | Average humidity exposure between 1-20 weeks of gestation. |
| a20 | Average AQI exposure between 1-20 weeks of gestation. |
| c_m_12 | Categroy of relative average temperature exposure between 1-12 weeks of preconception. 1: very cold, 2:moderate cold, 3:moderature temperature, 4: moderate hot, 5:bery hot. |
| c_r_12 | Categroy of relative average humidity exposure between 1-12 weeks of preconception. |
| c_a_12 | Categroy of relative average AQI exposure between 1-12 weeks of preconception. |
| c_m_4 | Categroy of relative average temperature exposure between 1-4 weeks of preconception. 1: very cold, 2:moderate cold, 3:moderature temperature, 4: moderate hot, 5:bery hot. |
| c_r_4 | Categroy of relative average humidity exposure between 1-4 weeks of preconception. |
| c_a_4 | Categroy of relative average AQI exposure between 1-4 weeks of preconception. |
| c_m14 | Categroy of relative average temperature exposure between 1-4 weeks of gestation.  1: very cold, 2:moderate cold, 3:moderature temperature, 4: moderate hot, 5:bery hot. |
| c_r14 | Categroy of relative average humidity exposure between 1-4 weeks of gestation. |
| c_a14 | Categroy of relative average AQI exposure between 1-4 weeks of gestation. |
| c_m512 | Categroy of relative average temperature exposure between 5-12 weeks of gestation.  1: very cold, 2:moderate cold, 3:moderature temperature, 4: moderate hot, 5:bery hot. |
| c_r512 | Categroy of relative average humidity exposure between 5-12 weeks of gestation. |
| c_a512 | Categroy of relative average AQI exposure between 5-12 weeks of gestation. |
| c_m1320 | Categroy of relative average temperature exposure between 13-20 weeks of gestation.  1: very cold, 2:moderate cold, 3:moderature temperature, 4: moderate hot, 5:bery hot. |
| c_r1320 | Categroy of relative average humidity exposure between 13-20 weeks of gestation. |
| c_a1320 | Categroy of relative average AQI exposure between 13-20 weeks of gestation. |
| c_m20 | Categroy of relative average temperature exposure between 1-20 weeks of gestation.  1: very cold, 2:moderate cold, 3:moderature temperature, 4: moderate hot, 5:bery hot. |
| c_r20 | Categroy of relative average humidity exposure between 1-20 weeks of gestation. |
| c_a20 | Categroy of relative average AQI exposure between 1-20 weeks of gestation. |
