# Supplementary material for: Association between ambient temperature and hypertensive disorders in pregnancy in China
Source: Nat Commun. 2020 Jun 10;11:2925. doi: 10.1038/s41467-020-16775-8 (PMC7286884; doi:10.1038/s41467-020-16775-8)
Supplement: Supplementary file 5 — Reporting Summary [file 41467_2020_16775_MOESM5_ESM.pdf]

nature research

Corresponding author(s): Dezhi Mu

Last updated by author(s): Mar 25, 2020

Reporting Summary

Nature Research wishes to improve the reproducibility of the work that we publish. This form provides structure for consistency and transparency in reporting. For further information on Nature Research policies, see Authors & Referees and the Editorial Policy Checklist.

Statistics

For all statistical analyses, confirm that the following items are present in the figure legend, table legend, main text, or Methods section.

n/a	Confirmed	
☒	☐	The exact sample size (*n*) for each experimental group/condition, given as a discrete number and unit of measurement
☒	☐	A statement on whether measurements were taken from distinct samples or whether the same sample was measured repeatedly
☐	☒	The statistical test(s) used AND whether they are one- or two-sided<br>*Only common tests should be described solely by name; describe more complex techniques in the Methods section.*
☐	☒	A description of all covariates tested
☒	☐	A description of any assumptions or corrections, such as tests of normality and adjustment for multiple comparisons
☐	☒	A full description of the statistical parameters including central tendency (e.g. means) or other basic estimates (e.g. regression coefficient) AND variation (e.g. standard deviation) or associated estimates of uncertainty (e.g. confidence intervals)
☒	☐	For null hypothesis testing, the test statistic (e.g. *F*, *t*, *r*) with confidence intervals, effect sizes, degrees of freedom and *P* value noted<br>*Give P values as exact values whenever suitable.*
☒	☐	For Bayesian analysis, information on the choice of priors and Markov chain Monte Carlo settings
☐	☒	For hierarchical and complex designs, identification of the appropriate level for tests and full reporting of outcomes
☒	☐	Estimates of effect sizes (e.g. Cohen's *d*, Pearson's *r*), indicating how they were calculated

Our web collection on statistics for biologists contains articles on many of the points above.

Software and code

Policy information about availability of computer code

Data collection	All statistical analyses were performed using SAS statistical software version 9.4 (SAS Institute Inc., NC, USA).
Data analysis	All statistical analyses were performed using SAS statistical software version 9.4 (SAS Institute Inc., NC, USA), Stata version 15.1 (Stata Corp., TX, USA), and R version 3.6.1.

For manuscripts utilizing custom algorithms or software that are central to the research but not yet described in published literature, software must be made available to editors/reviewers. We strongly encourage code deposition in a community repository (e.g. GitHub). See the Nature Research guidelines for submitting code & software for further information.

Data

Policy information about availability of data

All manuscripts must include a data availability statement. This statement should provide the following information, where applicable:
- Accession codes, unique identifiers, or web links for publicly available datasets
- A list of figures that have associated raw data
- A description of any restrictions on data availability

The dataset for this study was collected and generated from multiple meteorological and sociodemographic databases (with institutional restriction). The dataset is available from the corresponding authors upon reasonable request.

Field-specific reporting

Please select the one below that is the best fit for your research. If you are not sure, read the appropriate sections before making your selection.

☐ Life sciences ☐ Behavioural & social sciences ☒ Ecological, evolutionary & environmental sciences

For a reference copy of the document with all sections, see nature.com/documents/nr-reporting-summary-flat.pdf

Ecological, evolutionary & environmental sciences study design

All studies must disclose on these points even when the disclosure is negative.

Study description	Using a national cohort of 2,043,182 pregnant women in China, we evaluated the association between ambient temperature and HDP subgroups, including gestational hypertension, preeclampsia or eclampsia, and superimposed preeclampsia.
Research sample	Individual maternal data were collected from China's National Maternal Near Miss Surveillance System (NMNMSS). The NMNMSS collects the sociodemographic and obstetric information of pregnant and postpartum women from obstetric departments. The age range of participants was from 11 years to 60 years.
Sampling strategy	The sampling urban districts and rural counties of the National Maternal Near Miss Surveillance System (NMNMSS), which the surveillance health facilities located in, were based on China's National Maternal and Child Health Surveillance System (NMCHSS) and Provincial-level Maternal and Child Health Surveillance Systems (PMCHSSs). The surveillance sites of NMNMSS (326 urban districts and rural counties) were sampled randomly from combined NMCHSS and PMCHSSs within strata to ensure proportional representation of urban and rural populations across eastern, central, and western regions in China. Refer to the health facility selection criteria of WHO Global Survey for monitoring maternal and perinatal, once the surveillance sites are selected, two public health facilities located in these areas with more than 1000 deliveries per year are randomly selected. All the hospitals in Tibet were excluded due to the lack of skilled surveillance staff.<br>Since all the analysis were based on the multi-variable models, and the number of independent variables was less than 15, the sample size of the study (more than 2 millions) were exceeds the requirements (over 10-fold of independent variables).
Data collection	In each sampled health facility, data collection began when a pregnant woman was hospitalized in obstetrics department, until she was discharged from hospital or left obstetric department. An adapted individual survey form was used to collected information on sociodemographic characteristics, pregnancy complications and terminations, interventions and process indicators, and maternal and perinatal outcomes. This individual survey form was modified according to the WHO Multicountry Survey on Maternal and Newborn Health. Data were collected by obstetricians or nurses responsible for patient care mainly through the medical records review. When the individual survey form was completed, data were entered onto a web-based online reporting system centralized at the National Office for Maternal and Child Health Surveillance of China (NOMCHS).
Timing and spatial scale	The spatial scale was set by the distribution site of surveillance hospital. The surveillance hospitals in NMNMSS are located in 326 districts or counties throughout 30 provinces in mainland China, excluding Tibet.<br>The set of timing scale was based on following reasons: The Individual maternal data was based on delivery date of offsprings, which were from Jan 2012 to Dec 2017. The date of conception was calculated by the delivery date of offsprings according to their gestational age. Then, we counted 12 weeks before and in the first half of pregnancy to calculate the exposure duration (total 32 weeks). The complete meteorological data were available from Oct 2010 to Dec 2016. We matched the individual maternal data and meteorological data using exposure duration. Finally, the date of conception from Dec 2013 to Jul 2016 was considered in this study.
Data exclusions	We excluded pregnant women with i) other recorded obstetric complications (uterine rupture, placenta previa, abruption placentae, placental retention, uterine inertia and puerperal infection, abortion-related bleeding and infection) or ii) medical complications [heart disease, embolism, hepatopathy, severe anemia (hemoglobin concentration <70 g/L), renal disease (including urinary tract infection and chronic kidney disease), lung disease (including upper respiratory tract infection), diabetes (including gestational diabetes mellitus), HIV, desmosis, cancer, etc]. The exclusion criteria were pre-established.
Reproducibility	N/A. This study is an observational study.
Randomization	N/A. This study is an observational study.
Blinding	N/A. This study is an observational study.

Did the study involve field work? ☐ Yes ☒ No

Reporting for specific materials, systems and methods

We require information from authors about some types of materials, experimental systems and methods used in many studies. Here, indicate whether each material, system or method listed is relevant to your study. If you are not sure if a list item applies to your research, read the appropriate section before selecting a response.

Materials & experimental systems

n/a	Involved in the study	
☒	☐	Antibodies
☒	☐	Eukaryotic cell lines
☒	☐	Palaeontology
☒	☐	Animals and other organisms
☐	☒	Human research participants
☒	☐	Clinical data

Methods

n/a	Involved in the study	
☒	☐	ChIP-seq
☒	☐	Flow cytometry
☒	☐	MRI-based neuroimaging

Human research participants

Policy information about studies involving human research participants

Population characteristics

The sociodemographic characteristics of the included women are shown in Table 1. In total, 2,043,182 pregnant women were included during the study period. The median age is 28 years old (interquartile range 25-31 years old). In total, 1,973,919 pregnant women without complications (96.61%) served as controls. Among the 69,263 women with HDPs (3.39%), 23,704 women had gestational hypertension (1.16%), 38,166 women had preeclampsia or eclampsia (1.87%), 5,453 women had chronic hypertension (0.27%), and 1,940 women had superimposed preeclampsia (0.10%). Most women with HDPs were from level 2 and level 3 hospitals (level 2 and 3 represent the largest hospitals), had more than 4 antenatal care visits, were married, were in the 25-34 or 35-39 years old age groups, were nulliparous or with 1 parity, birthed singleton infants, did not birth small for gestational age (SGA) infants, and did not birth preterm infants. The women with HDPs tended to undergo a cesarean section, whereas the normotensive women tended to undergo vaginal delivery. The distributions of the region, fetus's sex and season of conception were similar in each group.

Recruitment

All patients who delivered in the surveillance hospitals were recruited. The possible bias was that the National Maternal Near Miss Surveillance System may oversampled large referral hospitals in urban districts, due to the difference between the population covered by the surveillance system and the whole Chinese maternal population.

Ethics oversight

No patients were directly involved in the design, recruitment, development, or interpretation of the study. The NMNMSS data was approved by the ethics committee of the West China Second University Hospital, Sichuan University, China (Protocol ID: 2012008).

Note that full information on the approval of the study protocol must also be provided in the manuscript.
